# A SCANO Nomogram for Individualized Prediction of the Probability of 1-Year Unfavorable Outcomes in Chinese Acute Ischemic Stroke Patients

**DOI:** 10.3389/fneur.2020.00531

**Published:** 2020-06-30

**Authors:** Xiang Li, Fusang Wang, Zhihong Zhao, Chao Sun, Jun Liao, Xuemei Li, Chaoping Huang, Linda Nyame, Zheng Zhao, Xiaohan Zheng, Junshan Zhou, Ming Li, Jianjun Zou

**Affiliations:** ^1^School of Basic Medicine and Clinical Pharmacy, China Pharmaceutical University, Nanjing, China; ^2^Department of Clinical Pharmacology, Nanjing First Hospital, Nanjing Medical University, Nanjing, China; ^3^Department of Neurology, The First Affiliated Hospital (People's Hospital of Hunan Province), Hunan Normal University, Changsha, China; ^4^Department of Neurology, Changsha Central Hospital, Changsha, China; ^5^Department of Neurology, Nanjing First Hospital, Nanjing Medical University, Nanjing, China; ^6^College of Life Science and Technology, China Pharmaceutical University, Nanjing, China

**Keywords:** nomogram, prediction, functional outcome, stroke, ischemic stroke

## Abstract

**Background and Purpose:** Accurate prediction of functional outcomes after stroke would provide evidence for reasonable poststroke management. This study aimed to develop and validate a nomogram for individualized prediction of 1-year unfavorable outcomes in Chinese acute ischemic stroke (AIS) patients.

**Methods:** We gathered AIS patients at the National Advanced Stroke Center of Nanjing First Hospital (China) between August 2014 and May 2017 within 12 h of symptom onset. The outcome measure was 1-year unfavorable outcomes (modified Rankin Scale 3–6). The patients were randomly stratified into the training (66.7%) and testing (33.3%) sets. With the training data, pre-established predictors were entered into a logistic regression model to generate the nomogram. Predictive performance of the nomogram model was evaluated in the testing data by calculating the area under the receiver operating characteristic curve (AUC-ROC), Brier score, and a calibration plot.

**Results:** A total of 807 patients were included into this study, and 262 (32.5%) of them had unfavorable outcomes. Systolic blood pressure, Creatinine, Age, National Institutes of Health Stroke Scale (NIHSS) score on admission, and fasting blood glucose were significantly associated with unfavorable outcomes and entered into the SCANO nomogram. The AUC-ROC of the SCANO nomogram in the testing set was 0.781 (Brier score: 0.166; calibration slope: 0.936; calibration intercept: 0.060).

**Conclusions:** The SCANO nomogram is developed and validated in Chinese AIS patients to firstly predict 1-year unfavorable outcomes, which is simple and convenient for the management of stroke patients.

## Introduction

Acute ischemic stroke (AIS) continues to be an important cause of morbidity and mortality worldwide, which can bring heavy economic burden for patients and their families (1, 2). Consequently, clinicians will be faced with a great challenge regarding unfavorable outcomes in these patients. Better identification of AIS patients with unfavorable outcomes could be useful to develop preventive strategies and reduce the risk of morbidity and mortality after stroke.

Some prognostic scores (3–5) and several nomograms (6–8) have identified some demographic and clinical characteristics to predict 3-month clinical outcomes for AIS patients. However, model for predicting 1-year unfavorable outcomes in AIS patients was not found. On the one hand, most of the functional improvements tend to be achieved during the first 3 months after stroke. Although the patients with AIS recover rapidly during the first few weeks, there are some additional recovery between 3 and 6 months (9, 10). On the other hand, between 3 and 12 months, one in six patients with AIS deteriorate in functional outcomes (11). Therefore, the models for predicting 3-month outcomes may be limited because these models could not be suitable for all patients. Furthermore, as the strongest predictors of 3-month unfavorable outcomes (3, 6, 12–14), the correlation of National Institutes of Health Stroke Scale (NIHSS) score with the outcomes varies with the time passed from the onset of cerebral ischemia (15). This also affects the predictive performance when these models were used to predict 1-year outcomes. As a result, these prognostic scores and nomograms cannot be extrapolated to predict 1-year unfavorable outcomes, and it is meaningful to establish a model to predict 1-year unfavorable outcomes.

Therefore, the present research aimed to develop and validate a nomogram by using a limited number of easily available variables to predict 1-year unfavorable outcomes for Chinese AIS patients.

## Methods

### Study Population

We conducted a retrospective analysis on the basis of data prospectively gathered from 1,831 consecutive AIS patients admitted at the National Advanced Stroke Center (NASC) of Nanjing First Hospital (China) from August 2014 to May 2017 within 12 h of symptom onset. We excluded patients with intracranial hemorrhage (ICH) on baseline brain computed tomography (CT) scan, lack of 1-year modified Rankin Scale (mRS) score, age <18 years, and NIHSS score on admission unknown for the present study. All variables with 20% missing values or more for further analysis were excluded.

All clinical, anamnestic, and demographic characteristics were recorded at the time of admission, including the following data: age, sex, NIHSS score on admission, systolic blood pressure (SBP), diastolic blood pressure, platelet count, international normalized ratio (INR), creatinine, fasting blood glucose (FBG), triglyceride (TG), low-density lipoprotein (LDL), glycated hemoglobin (HbA1c), medical history such as hypertension, and previous cerebral hemorrhage. Owing to the U-shape characteristic of SBP (16–18), SBP was divided into two groups: Group 1 with SBP between 100 and 180 mmHg and Group 2 with SBP <100 or >180 mmHg.

The unfavorable outcomes were defined as mRS 3–6, 1 year after stroke. Certified assessors evaluated the baseline NIHSS and 1-year mRS during a face to face or via telephone follow-up with the patients, their relatives, or their general practitioners.

### Statistical Analysis

The data were randomly stratified into the training and testing sets by the software package R version 3.5.2 (R Development Core Team, Auckland, New Zealand): two-thirds of the data were used for model development, whereas the remaining one-third were used to evaluate the performance of the model.

Continuous variables were represented as median value and interquartile range, and the differences between various groups were explored using the Mann–Whitney *U*-test. Categorical variables were instead expressed as number of events and percentage, dividing the number of events by the total number excluding missing and unknown cases. The differences between proportions were assessed by Fisher's exact test or the χ^2^ test, when appropriate.

To generate the nomogram, the variables with a probability value <0.10 in the univariate analysis or thought to be independent predictors of ischemic stroke were entered into a logistic regression model. A final model selection was carried out by a backward stepwise selection process with the Akaike information criterion. Regression coefficients and odds ratios (OR) with two-sided 95% confidence intervals (CIs) for each of the variable included in the model were finally calculated. Collinearity of combinations of variables that entered the multivariate logistic regression analysis was evaluated by the variation inflation factors (VIFs; <2 being considered nonsignificant) and condition index (<30 being considered nonsignificant).

The performance of the model was assessed in the testing data by discrimination (the ability of a proposed model to separate patients with different outcomes) and calibration (the relative distance of predictions from actual outcome). The discrimination of the nomogram model was assessed by the area under the receiver operating characteristic curve (AUC-ROC), whereas calibration was valued by the Hosmer–Lemeshow test, the Brier score, and a calibration plot. A 45° line indicates perfect calibration when the predictive value of the model perfectly matches the patient's actual risk. In all analyses, *p* < 0.05 was considered statistically significant.

The statistical analysis was performed using SPSS version 22.0 (IBM Corporation, Armonk, NY, USA) and Stata version 13.0 (StataCorp, College Station, TX, USA) statistical software. The software package R was used for model visualization.

## Results

After patients were excluded for NIHSS score on admission unknown (*n* = 143; 7.8%) and lack of 1-year mRS score (*n* = 881; 48.1%), 807 patients entered our study (median age 70 years; interquartile range: 62–79 years). The comparison of the characteristics of the patients between the included and excluded patients is shown in Table S1. The characteristics of the patients were well balanced between the training (*n* = 537, 66.5%) and testing (*n* = 270, 33.5%) sets (Table S2). The proportion of patients with unfavorable outcomes was 32.5% (262/807), and 10.5% (85/807) died within the follow-up period (mRS score = 6).

The clinical, anamnestic, demographic, and laboratory data of the patients in the favorable outcome cohorts (*n* = 545) and unfavorable outcome cohorts (*n* = 262) are shown in Table 1. Age, sex, NIHSS score on admission, SBP, INR, creatinine, FBG, TG, HbA1c, history of atrial fibrillation and smoking, endovascular therapy, and intravenous (IV) thrombolysis were found to be significant in the univariate analysis.

**Table 1:** Clinical, demographic, and laboratory data of study population stratified according to 1-year favorable or unfavorable outcome after acute ischemic stroke in Chinese patients.

	**Favorable outcome (mRS 0–2)**	**Unfavorable outcome (mRS 3–6)**	***P*-value**
Patients, *n*	545	262	
Age, years, median (IQR)	67 (59–75)	76 (67–82)	*P* = 0.000^†^^*^
Sex, *n* (%)			*P* = 0.000^*^
Male	398 (73.0)	159 (60.7)	
Female	147 (27.0)	103 (39.3)	
Medical history, *n* (%)			
Hypertension	387 (71.0)	194 (74.0)	*P* = 0.368
Hyperlipidemia	26 (4.8)	9 (3.4)	*P* = 0.383
Atrial fibrillation	48 (8.8)	56 (21.4)	*P* = 0.000^*^
Valvular heart disease	6 (1.1)	6 (2.3)	*P* = 0.191
Peripheral vascular disease	1 (0.2)	2 (0.8)	*P* = 0.516
Transient ischemic attack	4 (0.7)	0 (0)	*P* = 0.310
Previous cerebral infarction	105 (19.3)	60 (22.9)	*P* = 0.231
Previous cerebral hemorrhage	9 (1.7)	3 (1.1)	*P* = 0.806
Previous carotid endovascular treatment or endarterectomy	2 (0.4)	3 (1.1)	*P* = 0.401
Drinking, *n* (%)			*P* = 0.359
Never drinker	423 (77.6)	210 (80.2)	
Former drinker	25 (4.6)	15 (5.7)	
Current drinker	97 (17.8)	37 (14.1)	
Smoking, *n* (%)			*P* = 0.003^*^
Never smoker	376 (69.0)	191 (72.9)	
Former smoker	27 (5.0)	25 (9.5)	
Current smoker	142 (26.1)	46 (17.6)	
Baseline data			
NIHSS score on admission, median (IQR)	3 (1–5)	8 (3–14)	*P* = 0.000^†^^*^
SBP, *n* (%)			*P* = 0.006^*^
>100 and <180 mmHg	511 (93.8)	231 (88.2)	
≤100 or ≥180 mmHg	34 (6.2)	31 (11.8)	
DBP, mmHg, median (IQR)	84 (80–91)	82 (78–92)	*P* = 0.576^†^
Platelet count, 10^9^/L, median (IQR)	186 (156–223)	188 (148–223)	*P* = 0.660^†^
INR, median (IQR)	0.98 (0.94–1.04)	1.01 (0.97–1.07)	*P* = 0.000^†^^*^
Creatinine, umol/L, median (IQR)	73.30 (61.00–86.00)	80.60 (65.00–101.00)	*P* = 0.000^†^^*^
FBG, mmol/L, median (IQR)	5.23 (4.68–6.44)	6.06 (5.00–8.23)	*P* = 0.000^†^^*^
TC, mmol/L, median (IQR)	4.20 (3.61–4.89)	4.17 (3.45–4.98)	*P* = 0.494^†^
TG, mmol/L, median (IQR)	1.36 (0.97–1.92)	1.14 (0.83–1.62)	*P* = 0.000^†^^*^
LDL, mmol/l, median (IQR)	2.64 (2.10–3.23)	2.59 (2.04–3.09)	*P* = 0.191^†^
HbA1c, %, median (IQR)	5.88 (5.50–6.70)	6.10 (5.60–7.10)	*P* = 0.017^†^^*^
Endovascular therapy, *n* (%)	56 (10.3)	44 (16.8)	*P* = 0.007^*^
intravenous thrombolysis, *n* (%)	112 (20.6)	83 (31.7)	*P* = 0.000^*^

*mRS, indicates modified Rankin Scale; IQR, interquartile range; NIHSS, National Institutes of Health Stroke Scale; SBP, systolic blood pressure; DBP, diastolic blood pressure; INR, international normalized ratios; FBG, fasting blood glucose; TC, total cholesterol; TG, triglyceride; LDL, low density lipoprotein; HbA1c, Glycated hemoglobin*.

† *Calculated using Mann–Whitney U-test*.

* *included into the multiple logistic regression models*.

**Figure 1:**
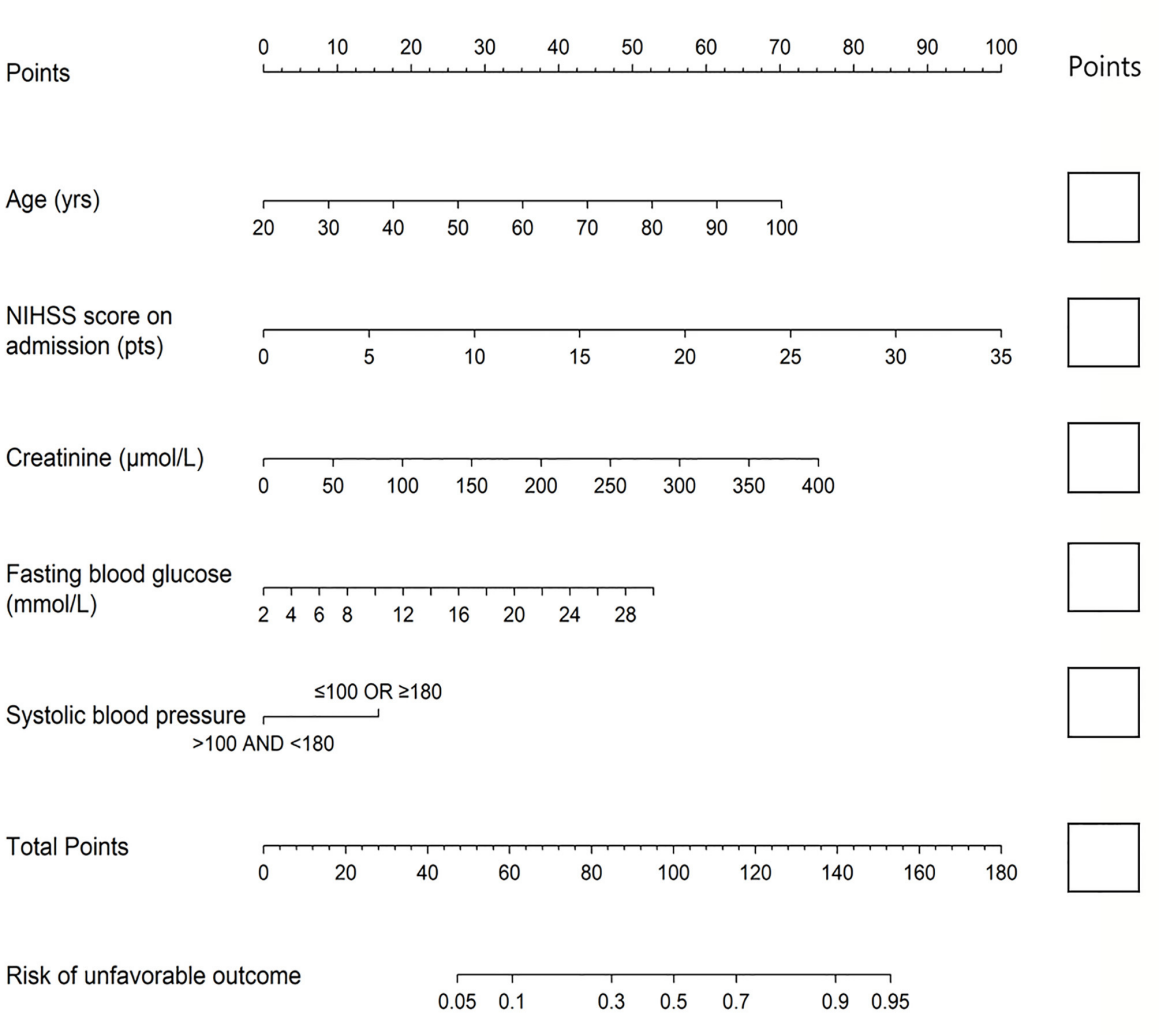
The nomogram used for predicting 1-year unfavorable outcomes in Chinese AIS patients. The final score (i.e., total points) is calculated as the sum of the individual score of each of the five variables included in the nomogram. AIS, acute ischemic stroke; NIHSS, National Institutes of Health Stroke Scale.

In multivariate analysis, age (OR: 1.050, *p* < 0.0001), NIHSS score on admission (OR: 1.172, *p* < 0.0001), creatinine (OR: 1.011, *p* = 0.003), FBG (OR: 1.111, *p* = 0.009), and SBP (OR: 2.375, *p* = 0.020) were finally entered into a logistic regression model to construct the SCANO nomogram for predicting the probability of unfavorable outcomes after the acute ischemic event (Table 2, Figure 1). No significant statistical collinearity was observed for any of the five pre-established variables that entered the multivariate logistic regression analysis. The logistic regression model resulted in the following: Log[*p*(*x*)/1–*p*(*x*)] = −6.763 + (0.049 × age) + (0.159 × NIHSS score on admission) + (0.105 × FBG) + (0.010 × creatinine) + (0.865 × SBP ≥ 180 or ≤ 100), where *p*(*x*) was the probability of 1-year unfavorable outcomes.

**Table 2:** Significant predictors of 1-year unfavorable outcome after acute ischemic stroke in Chinese patients.

	**OR**	**Error**	**Wald**	***P* value**	**95% CI**
Age	1.050	0.011	4.88	*P* = 0.000^*^	1.030–1.071
NIHSS score on admission	1.172	0.024	7.62	*P* = 0.000^*^	1.125–1.221
Creatinine	1.011	0.004	3.02	*P* = 0.003^*^	1.004–1.017
FBG	1.111	0.045	2.62	*P* = 0.009^*^	1.027–1.202
SBP	2.375	0.881	2.33	*P* = 0.020^*^	1.148–4.912

*NIHSS, indicates National Institutes of Health Stroke Scale; FBG, fasting blood glucose; SBP, systolic blood pressure; OR, odds ratio; CI, confidence interval*.

* *p < 0.05 was considered as significant in the multivariate logistic model*.

The nomogram was established by distributing a graphic preliminary score to each of the five predictors with a point range from 0 to 100, which was then summed to compute the total score and finally transited into an individual probability of 1-year unfavorable outcomes (from 5 to 95%). The lower total score of the nomogram indicated the lower likelihood of unfavorable outcomes, whereas the higher total score was linked with the higher likelihood of unfavorable outcomes. For example, a 60-year-old (about 35 points) stroke patient, with creatinine of 75 μmol/L (about 14 points) and FBG of 5 mmol/L (about 6 points), admitted with a NIHSS score of 3 (about 10 points) and SBP of 120 (0 points), would have a total nomogram score of 65 and <20% probability of unfavorable outcomes. Conversely, a 70-year- old (about 44 points) stroke patient, admitted with a NIHSS score of 15 (about 42.5 points), creatinine of 150 μmol/L (28 points), FBG of 12 mmol/L (18 points), and SBP of 185 (about 15 points), would have a total nomogram score of 147.5 and >90% probability of unfavorable outcomes.

**Figure 2:**
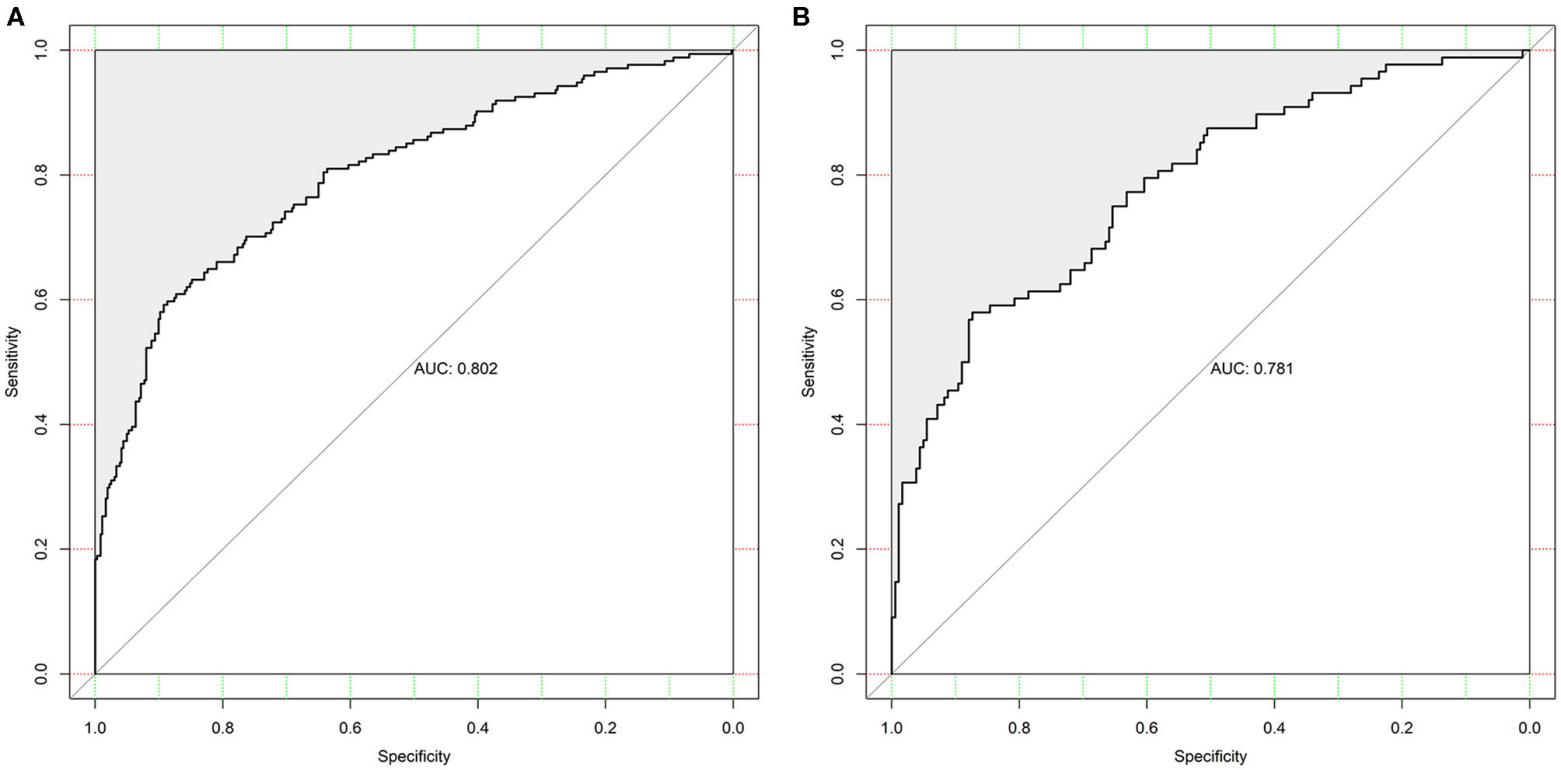
The ROC curve of the nomogram used for predicting 1-year unfavorable outcomes in Chinese AIS patients. **(A)** The ROC curve in the training set. **(B)** The ROC curves in the testing set. ROC, receiver operating characteristic; AIS, acute ischemic stroke.

**Figure 3:**
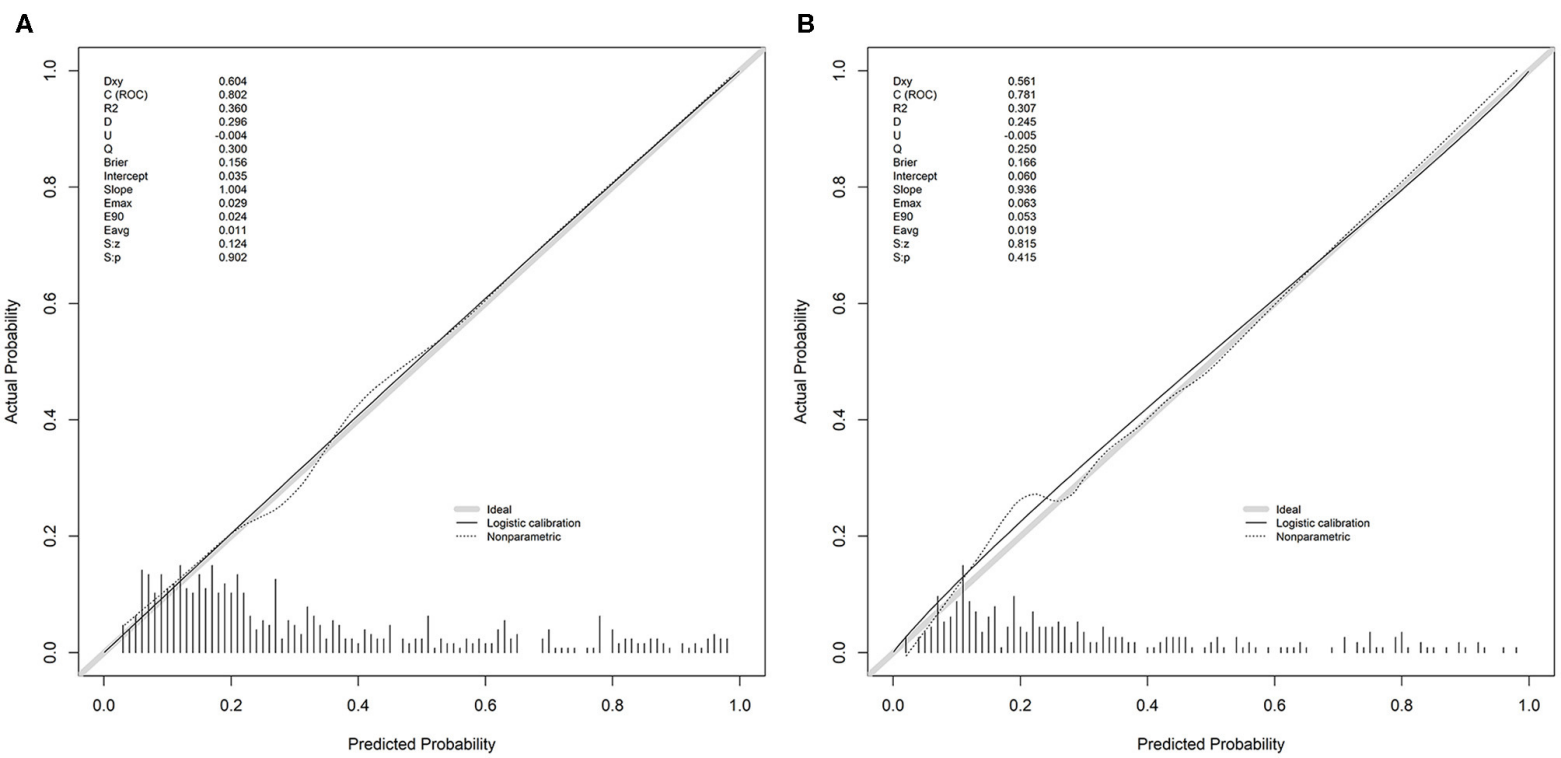
The calibration plot for the nomogram used for predicting 1-year unfavorable outcomes in Chinese AIS patients. **(A)** The calibration plot in the training set. **(B)** The calibration plot in the testing set. Dashed line is reference line where an ideal nomogram would lie. The dotted line is the performance of nomogram, whereas the solid line corrects for any bias in nomogram. AIS, acute ischemic stroke.

The discrimination of the SCANO nomogram was good, with an AUC–ROC of 0.802 (95% CI: 0.761–0.843) in the training set (Figure 2A) and 0.781 (95% CI: 0.721–0.840) in the testing set (Figure 2B). The Hosmer–Lemeshow goodness-of-fit test showed good calibration of the nomogram in the training (*p* = 0.437) and testing (*p* = 0.178) sets. The calibration plot revealed adequate fit of the model predicting the risk of 1-year unfavorable outcomes in the training (Figure 3A) and testing (Figure 3B) sets. The calibration slope, calibration intercept, and Brier score in the testing data were 0.936, 0.060, and 0.166, respectively.

In the testing data, there were 39 (14.4%) patients with a risk probability <10%, and only two of these had an unfavorable outcome (0.21 specificity, 0.97 sensitivity, 0.37 positive predictive value, and 0.93 negative predictive value). The number of a risk probability <40% was 198 (73.3%), 38 of whom (19.2%) had unfavorable outcomes (0.88 specificity, 0.60 sensitivity, 0.70 positive predictive value, and 0.82 negative predictive value). Finally, the patients with a high-risk probability (i.e., >80%) were 18/270 (6.7%), the vast majority of whom (16/18; 88.9%) had an unfavorable prognosis (0.99 specificity, 0.24 sensitivity, 0.91 positive predictive value, and 0.73 negative predictive value).

## Discussion

Stroke is a major cause of death and lifelong disability worldwide (1, 2). As a public health problem, early prediction of unfavorable outcomes of AIS patients should be an important reference for accurate clinical and therapeutic management and has the potential to enhance clinical care and rehabilitation. So far, there are some reports on 3-month unfavorable outcomes after stroke (4, 5, 19, 20), and previous studies have attempted to use the nomogram to predict 3-month unfavorable outcomes for AIS patients (6–8). However, to date, there is no nomogram to predict unfavorable outcomes in the longer term, and the SCANO nomogram was developed and validated in Chinese AIS patients to firstly predict 1-year unfavorable outcomes, which is advantageous by providing individualized risk assessment in a user-friendly and dynamic manner. Furthermore, we emphasize that we have included only the five common variables, which are available in clinical practice. In addition, our nomogram may be more easily and quickly employed in a clinical practice if used with related software on a computer or a handheld device.

Our study showed that age, creatinine, FBG, NIHSS score on admission, and SBP were significantly independent predictors of unfavorable outcomes in Chinese AIS patients. Firstly, consistent with previous studies (4, 6, 12), our study showed that age and NIHSS score on admission were significant and independent predictors of unfavorable outcomes in Chinese AIS patients. Indeed, the NIHSS score manifests a more severe stroke, which can be more likely associated with unfavorable outcomes, whereas age can generally bring about a less intense recovery. Secondly, serum creatinine concentration is generally considered as an index of renal function. However, the relationship between renal function and stroke outcomes is controversial (21, 22). Future studies regarding to the mechanisms of creatinine in the pathogenesis of stroke are needed. Thirdly, the results of our study showed a relationship between admission SBP and unfavorable outcomes, which was described as a distinct U-shaped relation in the previous studies (16–18). Finally, hyperglycemia in ischemic stroke patients could predict unfavorable outcomes independently, possibly owing to increased blood–brain barrier disruption with higher hemorrhagic risk (23, 24) or increased lactic acid production in ischemic tissue leading to a greater infarct size (25).

Consequently, we have developed and validated a nomogram on the basis of the combination of these five variables, and we observed relatively high predictive accuracy (AUC, 0.781) of unfavorable outcomes. Importantly, a risk probability > 80% derived from the nomogram displayed a remarkably positive predictive value (i.e., 0.91), which can predict accurately unfavorable outcomes. On the other hand, a risk probability <10% was associated with 0.93 negative predictive value, thus enabling to exclude accurately the possibility of developing unfavorable outcomes. Therefore, the SCANO nomogram may provide more detailed information to facilitate the early identification of patients with high probability of unfavorable outcomes and to discuss prognosis with patients and their families.

There are some limitations that might have an impact on the interpretation of our results. Firstly, this is characterized by the inherent disadvantage of any retrospective single-center study such as collection and entry bias, and possible residual confounding. For example, there were some cases that were lost to follow-up. It is unclear whether we have overestimated or underestimated the unfavorable outcomes after AIS. However, most of models to predict functional outcomes of ischemic stroke were built in this way. Secondly, patient populations with limited geographical or ethnicity area have been a concern in the development of predictive models, and racial differences may have affected the unfavorable outcomes (26). External validation, especially in different populations, is the future work for this predictive model. Finally, data of known neurobiological predictors such as infarct size (27) were not available in our study. Notwithstanding these limitations, to our knowledge, the present study is the first attempt to develop and validate a nomogram to predict 1-year unfavorable outcomes in a cohort of AIS patients in agreement with the current guidelines.

## Conclusion

The SCANO nomogram may be a reliable and easy-to-use tool to firstly predict 1-year unfavorable outcomes, which is developed and validated in Chinese AIS patients. External validations are needed to ensure its value in predicting the 1-year outcomes for AIS patients.

## Data Availability Statement

The datasets generated for this study are available on request to the corresponding author.

## Ethics Statement

All patients have given their written informed consent and the scientific use of the data obtained from the Nanjing First Hospital Stroke Registry was approved by the Ethics Committees of Nanjing First Hospital in accord with the Helsinki declaration and internal protocol.

## Author Contributions

ML and JZo conceived, designed, and supervised the study. FW, CS, XuL, CH, and JZh acquired the data. XiL and ZhiZ analyzed and interpreted the data, provided statistical analysis, had full access to all of the data in the study, and are responsible for the integrity of the data and the accuracy of the data analysis. XiL, LN, and ZheZ drafted the manuscript. JZo, ML, ZhiZ, and JL critically revised the manuscript for important intellectual content. All authors contributed to the article and approved the submitted version.

## Conflict of Interest

The authors declare that the research was conducted in the absence of any commercial or financial relationships that could be construed as a potential conflict of interest.
