# Corrigendum: A SCANO Nomogram for Individualized Prediction of the Probability of 1-Year Unfavorable Outcomes in Chinese Acute Ischemic Stroke Patients

**DOI:** 10.3389/fneur.2020.00837

**Published:** 2020-08-14

**Authors:** Xiang Li, Fusang Wang, Zhihong Zhao, Chao Sun, Jun Liao, Xuemei Li, Chaoping Huang, Linda Nyame, Zheng Zhao, Xiaohan Zheng, Junshan Zhou, Ming Li, Jianjun Zou

**Affiliations:** ^1^School of Basic Medicine and Clinical Pharmacy, China Pharmaceutical University, Nanjing, China; ^2^Department of Clinical Pharmacology, Nanjing First Hospital, Nanjing Medical University, Nanjing, China; ^3^Department of Neurology, The First Affiliated Hospital (People's Hospital of Hunan Province), Hunan Normal University, Changsha, China; ^4^Department of Neurology, Changsha Central Hospital, Changsha, China; ^5^Department of Neurology, Nanjing First Hospital, Nanjing Medical University, Nanjing, China; ^6^College of Life Science and Technology, China Pharmaceutical University, Nanjing, China

**Keywords:** nomogram, prediction, functional outcome, stroke, ischemic stroke

In the published article there was an error regarding the affiliation for Jianjun Zou and Zheng Zhao. Instead of having affiliation 1 and affiliation 2, Jianjun Zou and Zheng Zhao have only affiliation 2.

The authors apologize for this error and state that this does not change the scientific conclusions of the article in any way. The original article has been updated.

