# Supplementary material for: A SCANO Nomogram for Individualized Prediction of the Probability of 1-Year Unfavorable Outcomes in Chinese Acute Ischemic Stroke Patients
Source: Front Neurol. 2020 Jun 30;11:531. doi: 10.3389/fneur.2020.00531 (PMC7338753; doi:10.3389/fneur.2020.00531)
Supplement: Supplementary file 1 [file Data_Sheet_1.docx]

**TABLE S1 |** Demographics and clinical characteristics of the patients excluded and included.

|  | **Excluded patients**  **(n=1024)** | **Included patients**  **(n=807)** | ***P*-value** |
| --- | --- | --- | --- |
| Unfavorable outcome at 3 months, n (%) | 14(25.0) | 262(32.5) | P = 0.247 |
| Age, years, median (IQR) | 69(60-78) | 70(62-79) | P = 0.193 |
| Male Sex, n (%) | 692(67.6) | 557(69.0) | P = 0.510 |
| Medical history, n (%) |  |  |  |
| Hypertension | 729(71.7) | 581(72.0) | P = 0.882 |
| Hyperlipidemia | 23(2.3) | 35(4.3) | P = 0.012* |
| Atrial fibrillation | 131(12.9) | 104(12.9) | P = 0.997 |
| Valvular heart disease | 15(1.5) | 12(1.5) | P = 0.983 |
| Peripheral vascular disease | 4(0.4) | 3(0.4) | P = 0.941 |
| Transient ischemic attack | 4(0.4) | 4(0.5) | P = 1.000 |
| Previous cerebral infarction | 212(20.8) | 165(20.4) | P = 0.843 |
| Previous cerebral hemorrhage | 21(2.1) | 12(1.5) | P = 0.355 |
| Previous carotid endovascular treatment or endarterectomy | 2(0.2) | 5(0.3) | P = 0.901 |
| Drinking, n (%) |  |  | P = 0.000* |
| Never drinker | 657(65.2) | 633(78.4) |  |
| Former drinker | 94(9.3) | 40(5.0) |  |
| Current drinker | 256(25.4) | 134(16.6) |  |
| Smoking, n (%) |  |  | P = 0.000* |
| Never smoker | 561(55.1) | 567(70.3) |  |
| Former smoker | 109(10.7) | 52(6.4) |  |
| Current smoker | 348(34.2) | 188(23.3) |  |
| Baseline data |  |  |  |
| NIHSS score on admission, median (IQR) | 4(2-8) | 3(2-8) | P = 0.091 |
| SBP, n (%) |  |  | P = 0.939 |
| >100 and <180 mmHg | 937(92.0) | 742(91.9) |  |
| ≤100 or ≥180 mmHg | 81(8.0) | 65(8.1) |  |
| DBP, mmHg, median (IQR) | 82(80-93) | 83(80-91) | P = 0.559 |
| Platelet count, 10^9^/L, median (IQR) | 192(158-229) | 187(154-223) | P = 0.068 |
| INR, median (IQR) | 0.98(0.93-1.04) | 1.00(0.95-1.05) | P = 0.688 |
| Creatinine, umol/L, median (IQR) | 75.00(62.00-90.00) | 76.00(62.00-90.00) | P = 0.725 |
| FBG, mmol/L, median (IQR) | 5.34(4.69-7.00) | 5.39(4.74-6.95) | P = 0.638 |
| TC, mmol/L, median (IQR) | 4.25(3.58-5.01) | 4.20(3.55-4.94) | P = 0.916 |
| TG, mmol/L, median (IQR) | 1.27(0.91-1.85) | 1.27(0.92-1.84) | P = 0.380 |
| LDL, mmol/l, median (IQR) | 2.63(2.04-3.26) | 2.62(2.09-3.18) | P = 0.787 |
| HbA1c, %, median (IQR) | 5.90(5.50-6.70) | 5.92(5.50-6.80) | P = 0.397 |
| Endovascular therapy | 135(13.2) | 100(12.4) | P = 0.615 |
| Intravenous thrombolysis, n (%) | 200(19.5) | 195(24.2) | P = 0.017* |

*mRS, indicates modified Rankin Scale; NIHSS, National Institutes of Health Stroke Scale;* S*BP, s*ystolic *blood pressure; DBP,* diastolic *blood pressure; INR, international normalized ratios; FBG, fasting blood glucose; TC, total cholesterol; TG, triglyceride; LDL, low density lipoprotein; HbA1c, Glycated hemoglobin; IQR: interquartile range.*

**TABLE S2 |** Characteristics of the training and testing set.

|  | **Training set**  **(n=537)** | **Testing set**  **(n=270)** | ***P*-value** |
| --- | --- | --- | --- |
| Unfavorable outcome at 1 year, n (%) | 174(32.4) | 88(32.6) | P = 0.957 |
| Age, years, median (IQR) | 70(61-78) | 69(61-79) | P = 0.878 |
| Male Sex, n (%) | 365(68.0) | 192(71.1) | P = 0.363 |
| Medical history, n (%) |  |  |  |
| Hypertension | 382(71.1) | 199(73.7) | P = 0.443 |
| Hyperlipidemia | 23(4.3) | 12(4.4) | P = 0.915 |
| Atrial fibrillation | 66(12.3) | 38(14.1) | P = 0.476 |
| Valvular heart disease | 9(1.7) | 3(1.1) | P = 0.532 |
| Peripheral vascular disease | 2(0.4) | 1(0.4) | P = 0.996 |
| Transient ischemic attack | 4(0.7) | 0(0) | P = 0.373 |
| Previous cerebral infarction | 98(18.2) | 67(24.8) | P = 0.029* |
| Previous cerebral hemorrhage | 4(0.7) | 8(3.0) | P = 0.032* |
| Previous carotid endovascular treatment or endarterectomy | 4(0.7) | 1(0.4) | P = 0.869 |
| Drinking, n (%) |  |  | P = 0.967 |
| Never drinker | 421(78.4) | 212(78.5) |  |
| Former drinker | 26(4.8) | 14(5.2) |  |
| Current drinker | 90(16.8) | 44(16.3) |  |
| Smoking, n (%) |  |  | P = 0.804 |
| Never smoker | 376(70.0) | 191(70.7) |  |
| Former smoker | 33(6.1) | 19(7.0) |  |
| Current smoker | 128(23.8) | 60(22.2) |  |
| Baseline data |  |  |  |
| NIHSS score on admission, median (IQR) | 3(2-8) | 3(2-8) | P = 0.817 |
| SBP, n (%) |  |  | P = 0.115 |
| >100 and <180 mmHg | 488(90.9) | 254(94.1) |  |
| ≤100 or ≥180 mmHg | 49(9.1) | 16(5.9) |  |
| DBP, mmHg, median (IQR) | 82(80-90) | 85(79-93) | P = 0.611 |
| Platelet count, 10^9^/L, median (IQR) | 186(151-221) | 190(160-226) | P = 0.209 |
| INR, median (IQR) | 1.00(0.95-1.05) | 0.99(0.95-1.06) | P = 0.886 |
| Creatinine, umol/L, median (IQR) | 77.00(62.00-91.00) | 74.00(62.00-86.00) | P = 0.217 |
| FBG, mmol/L, median (IQR) | 5.41(4.74-6.94) | 5.37(4.75-7.06) | P = 0.985 |
| TC, mmol/L, median (IQR) | 4.25(3.63-4.94) | 4.08(3.35-4.91) | P = 0.015* |
| TG, mmol/L, median (IQR) | 1.31(0.94-1.90) | 1.23(0.88-1.69) | P = 0.055 |
| LDL, mmol/l, median (IQR) | 2.64(2.14-3.18) | 2.58(1.86-3.17) | P = 0.098 |
| HbA1c, %, median (IQR) | 5.90(5.50-6.80) | 6.00(5.60-6.90) | P = 0.418 |
| Endovascular therapy, n (%) | 67(12.5) | 33(12.2) | P = 0.829 |
| Intravenous thrombolysis, n (%) | 131(24.4) | 64(23.7) | P = 0.918 |
